# Exposure to polystyrene nanoparticles leads to dysfunction in DNA repair mechanisms in Caco-2 cells

**DOI:** 10.1186/s40659-025-00629-y

**Published:** 2025-07-11

**Authors:** Agata Kustra, Mirosław Zając, Piotr Bednarczyk, Kamila Maliszewska-Olejniczak

**Affiliations:** Department of Physics and Biophysics, Institute of Biology, Warsaw University of Life Sciences– SGGW, Warsaw, Poland

**Keywords:** Polystyrene nanoparticles, DNA damage response, DNA-DSBs, DNA-SSBs, Apoptosis, PARP

## Abstract

**Background:**

Recent studies have highlighted the critical health implications of environmental exposure to nanoplastics, particularly concerning their effects on human gastrointestinal cells. In this study, we used human colorectal adenocarcinoma (Caco-2) cells to investigate the exposure of polystyrene nanoparticles (PNPs) to cellular processes and DNA repair.

**Methods:**

We exposed Caco-2 cells to various concentrations of PNPs and monitored cytotoxicity, ROS levels, PARP-1-dependent apoptosis, DNA damage, and changes in DNA damage response (DDR) gene expression.

**Results:**

The results indicated that although PNPs did not directly cause SSBs or DSBs, as evidenced by comet assays and γH2AX staining, they induced oxidative stress and significantly altered the expression of genes required for DDR. In particular, critical genes involved in the base excision repair (BER) pathway and DSBs repair were downregulated, suggesting a potential impairment of the cell’s ability to repair oxidative DNA damage.

**Conclusions:**

This study highlights the sublethal effects of nanoplastics on intestinal barrier cells. It underscores the possible risks of exposure to these environmental contaminants, which can lead to genome instability and other long-term health consequences.

## Introduction

Over the last few years, the growing concern about environmental pollution has brought nanoplastics to the forefront of research, particularly their impact on human health [1]. Nanoplastics have been defined as particles that are unintentionally generated, either through the degradation or manufacturing of plastic materials, exhibiting colloidal behavior and falling within the size range of 1 to 1000 nm [2]. They are now recognized as contaminants in various ecosystems, including aquatic environments and the food chain [2]. Several studies have identified potential toxicological effects associated with the bioaccumulation of nanoplastics in human tissues [3]. Nanoplastics can induce oxidative stress, inflammation, and cellular damage through their ability to penetrate biological membranes and interact with cellular structures [4]. Moreover, the small size of nanoplastics allows them to bypass physical barriers in the body, leading to their internalization and interaction with intracellular components such as DNA, proteins, and lipids [5]. Particles less than 1.5 μm in diameter could be transported to deeper tissues [6]. Nanoplastics can accumulate in cells and tissues of the gastrointestinal tract, respiratory, reproductive, or nervous systems. The surface charge of plastic particles can vary depending on the type of polymer and environmental conditions. Plastic particles are inherently hydrophobic due to their non-polar nature. This hydrophobicity is a key factor in their interaction with other materials and their behavior in aqueous environments [7]. Moreover, as the radius of a polystyrene sphere decreases, the surface area-to-volume ratio increases. These all characteristics increase the ability to use various mechanisms to cross cell membrane barriers and tissue epithelia to enter the bloodstream and lymphatic system, allowing them to be transported over long distances [8].

Exposure to nanoplastics can lead to harmful effects, including genotoxicity, which can directly interact with DNA, altering the base excision repair (BER) or indirectly *via* oxidative stress, inflammation, and apoptosis [1, 6, 8]. BER corrects DNA damage caused by oxidation, deamination, and alkylation with minimal disruption to the DNA structure [9]. This process occurs efficiently without energy input and functions in nuclear and mitochondrial DNA, which prevents cancer, aging, and neurodegenerative diseases. Many studies indicate that nanoplastics induce intracellular reactive oxygen species (ROS) production. Membrane lipids are oxidized by ROS, which results in aldehydes that chemically change DNA bases. These modified bases can accumulate in human tissues or undergo changes associated with aging and disease. Oxidative damage is caused by excess reactive oxygen species accumulating, affecting mitochondria, lipids, proteins, RNA, and DNA [10]. However, most research on nanoplastics and oxidative DNA damage has yielded inconclusive results [1]. In addition to oxidative stress, direct interactions between nanoplastics and DNA, as well as the release of additives from nanoplastics, may play a more significant role in genotoxicity.

 [1]. Despite these concerns, the full scope of nanoplastics’ impact on human DNA, health, and the environment still needs to be completely understood, and further investigation is needed to determine the long-term consequences of nanoplastics’ accumulation within biological systems.

In the present study, our objective was to explore how human Caco-2 intestinal barrier cells respond after exposure to polystyrene nanoparticles (PNPs), particularly concerning the DNA repair mechanisms and cellular stress responses. The findings offer important insights into the biological mechanisms that influence the effects of nanoplastics in vitro. We used the Caco-2 cells as a physiological model to simulate the intestinal epithelial barrier, enabling us to study the interaction of nanoplastics with the digestive system and to investigate the associated disruption in DNA repair mechanisms and cellular adaptations.

## Materials and methods

### Sample of polystyrene nanoparticles

The PNPs were purchased from Sigma-Aldrich (cat. no. 43302, St. Louis, MO, USA). An aqueous suspension (10% WT) of the sample with an average diameter of 100 nm and a density of 1.05 g/cm^3^ was used for the study. The PNPs suspension was stored at 2–8 °C. A standardized sample was used in the survey since the characterization of these particles showed that they were homogeneous and of the same size (100 nm) and consisted of poly[(divinylbenzene)styrene]. The standardized sample of PNPs was previously defined by their spherical shape, uniform surface, and synthetic polymeric composition, as confirmed by Scanning Electron Microscopy (SEM) and Energy Dispersive X-ray Spectroscopy (EDS) [8]. Their chemical identity was further validated by Fourier Transform Infrared Spectroscopy (FTIR), confirming the presence of characteristic polystyrene functional groups that influence their physicochemical properties, hydrophobicity, and potential for biomolecular interactions.

### Cell culture and treatments

The colon adenocarcinoma cell line (Caco-2) from Sigma-Aldrich was used in the experiments. In Dulbecco’s minimal essential medium (DMEM) (Sigma-Aldrich, USA), Caco-2 was cultivated using a concentrate of non-essential amino acids (10 mg/mL) (NEAA) (Sigma-Aldrich, USA), 10% fetal bovine serum (Gibco, USA), and penicillin and streptomycin (10 mg/mL) (Sigma-Aldrich, USA). Cells were grown in an environment with 95% humidity, 5% CO_2_, and 37 °C. Twice a week, cells were passaged at 70–80% confluence and treated with 0.25% trypsin-EDTA solution (Sigma-Aldrich, St. Louis, MO, USA) to sustain cell culture.

### The Trypan blue assay

The viability of Caco-2 cells was assessed using 0.4% trypan blue solution (Bio-Rad). The cells were mixed with trypan blue (1:1 ratio– 10 µl of Caco- 2 cells + 10 µl of trypan blue stain). Before treatment, the cells were seeded on T-25 flasks, and were treated with trypan blue before they exceeded 70% confluence. Cell viability was evaluated after the cells were treated with PNPs concentrations of 0, 50, 100, 400, 800, and 1200 µg/mL. Cells were incubated for 3 h, 24 h and 48 h. Three biological replicates were performed for each concentration and incubation time. In two technical replicates, measurements were carried out using a TC20™ Automated Cell Counter (Bio-Rad, USA) on slides dedicated to this device. Cell viability in the test population was expressed as the content of viable cells (%) in the test cell population.

### The clonogenic assay

After 24-hour treatment with the PNPs, cells were seeded in sufficient numbers to produce colonies within 1–3 weeks. A minimum of 50 cells was considered a single colony, and an assay was performed as published before [11–13]. Caco-2 cells were then seeded at 300,000 cells/well in 6-well plates in 2 mLDMEM, in at least three technical replicates, and after 24 h, the cells were treated with PNPs at concentrations of 0, 50, 100, 400, 800, and 1200 µg/mL. After another 24 h of exposure, 1,000 cells/well were seeded in 2 mL of fresh DMEM medium to form colonies and incubated at 37 °C / 5% CO_2_. After nine days, the cells were fixed and stained with Coomassie Brilliant Blue R-250 dye (Bio-Rad) (2 mL/well), incubating for 10–15 min at RT. The Coomassie solution was then collected and washed with the Coomassie Brilliant Blue R-250 wash-off solution (Bio-Rad). The residual buffer was removed by immersing the plates in warm tap water. Readings were performed the following day. Stained colonies were counted for individual images (of each well of a 6-well plate) saved in TIFF format. The colonies were counted using the automated countPHICS software [14]. The collected data on the number of colonies formed were then used to plot the survival curve.

### Measurement of reactive oxygen species

A fluorescent DCFDA (2’, 7’-dichlorofluorescein acetate) probe was used to measure the level of reactive oxygen species in Caco-2 cells as described previously [15]. On the first day, the 96-well plate was treated with collagen diluted with 1:10 deionized water (Sigma-Aldrich) to prevent Caco-2 colon adenocarcinoma cells from detaching. On the next day, 50,000 cells per well were seeded. On the third day, cells were treated with PNPs at 0, 50, 100, 400, 800, and 1200 µg/mL concentrations in at least three technical replicates and 1.5 mM of H_2_O_2_ (Sigma-Aldrich) as a positive control. After 24 h, the cells were washed with PBS (VWR), followed by incubation with 10 µM DCFDA (Sigma-Aldrich, USA) in HBSS (Sigma-Aldrich) (100 µl per well) for 3 h at 37 °C, 5% CO_2_. Fluorescence intensity was measured using a Fluoroskan ASCENT microplate reader (Thermo Fisher Scientific, USA) at 485 nm/520 nm wavelengths.

### Flow cytometric analysis of cycling cell populations

Analysis of cell cycle phases in the Caco-2 line after exposure to PNPs was performed by flow cytometry technique using immunofluorescence staining of cells that had incorporated bromodeoxyuridine (BrdU) using an Apoptosis, DNA Damage and Cell Proliferation Kit (BD Pharmingen™), as published before [13]. For the experiment, 1 × 10^6^ Caco-2 cells were prepared after 24-hour incubation with 100 µg/mL PNPs and 2-hour incubation with etoposide (Sigma-Aldrich), as a positive control with a final concentration of 50 µM. Cells were washed with PBS solution (VWR) and then treated with 1 mM BrdU from BD Pharmingen™ kit. Incubation was carried out for 60 min. at 37 °C, 5% CO_2_. were collected by trypsinization, washed with 1% BSA/PBS (Sigma-Aldrich), fixed and permeabilized with BD Cytofix/Cytoperm™ Fixation/Permeabilization Solution, BD Cytofix/Cytoperm™ Plus Permeabilization Buffer (BD Pharmingen™) and stained with PerCP-Cy5.5 Mouse Anti-BrdU Antibody (BD Pharmingen™) at 5 µl/sample. After a 20-minute incubation at RT, staining was performed with 1 µg/mL DAPI (BD Pharmingen™) diluted in 1% BSA/PBS. Analysis was performed using the BD™ LSR II flow cytometer equipped with FACSdiva software. Each experiment was conducted on different cell batches. The data were presented as an original multiparameter cell cycle analysis (G0-G1, S, G2-M).

### Quantification of apoptotic cells dependent on PARP-1 cleavage

1 × 10^6^ Caco-2 cells were prepared after 24-hour incubation with 100 µg/mL PNPs and 2-hour incubation with etoposide (Sigma-Aldrich), as a positive control with a final concentration of 50 µM. Cells were washed with PBS solution (VWR) and then treated with 100 µl of 1 mM BrdU from BD Pharmingen™ kit. Incubation was carried out for 60 min in a cell incubator at 37 °C, 5% CO_2_. Then cells were collected by trypsinization, washed with 1% BSA/PBS (Sigma-Aldrich), fixed and permeabilized with BD Cytofix/Cytoperm™ Fixation/Permeabilization Solution, BD Cytofix/Cytoperm™ Plus Permeabilization Buffer (BD Pharmingen™) and stained with PE Mouse Anti-Cleaved PARP (Asp214) antibody (BD Pharmingen™) in a 5 µl/sample volume. After a 20-minute incubation at room temperature, additional staining was performed with 1 µg/mL DAPI (BD Pharmingen™) diluted in 1% BSA/PBS. An assay was performed using the BD™ LSR II flow cytometer equipped with FACSdiva. Each experiment was performed on different cell batches.

### Alkaline comet assay

For the experiment, 1 × 10^5^ Caco-2 cells grown on T-75 flasks, were prepared after a 24-hour incubation with 100 µg/mL PNPs and a 2-hour incubation with etoposide (Sigma-Aldrich), a positive control with a final concentration of 50 µM. To 500 µl of dissolved agarose (Bio-techne, R&D Systems) was added 50 µl of cells immersed in chilled 1 mL PBS (VWR), then applied 50 µl each to two CometSlide wells (Bio-techne, R&D Systems) and spread over the entire surface. The slides were placed in the dark for 10 min at 2–8ºC. The next step was to subject the cells to lysis by immersing the slides in lysis solution (Bio-techne, R&D Systems) for 60 min at 4 °C. The samples were then incubated in an alkaline unwinding solution (pH > 13) (NaOH pellets 0.4 g; 200 mM EDTA, pH 10 250 µl; H_2_O 49.75 mL) for 20 min at RT (in the dark). Electrophoresis was carried out in a cooled Comet Assay Tank (Cleaver Scientific), immersing the slides in an alkaline buffer, pH 13 (NaOH granules 8 g; 500 mM EDTA 2 mL; H_2_O 1 L). After electrophoresis, the samples were washed, fixed with 70% ethanol (POCH), and thoroughly dried. After fixation, cells were stained by applying 100 µl of diluted SYBR Safe solution (Invitrogen) to each sample for 30 min in the dark. The samples were dried and analyzed under a Leica TCS SP5 confocal microscope (Leica, Germany) at 502/530 nm wavelengths. The comet assay images were analyzed (% of DNA in head and % of DNA in tail) using OpenComet v1.3.1 software as described previously [16]. For each treatment group, at least 50 comets were scored per biological replicate. Three independent experiments (*n* = 3) were performed.

### Flow cytometry analysis of DNA double-strand breaks

1 × 10^6^ Caco-2 cells grown on T-75 flasks were prepared after 24-hour incubation with 100 µg/mL PNPs and 2-hour incubation with 50 µM etoposide (Sigma-Aldrich), as a positive control. Cells were washed with PBS solution (VWR) and then treated with 1 mM BrdU from BD Pharmingen™ kit. Incubation was carried out for 60 min. at 37 °C in 5% CO_2_. Then cells were collected by trypsinization, washed with 1% BSA/PBS (Sigma-Aldrich), fixed and permeabilized with BD Cytofix/Cytoperm™ Fixation/Permeabilization Solution, BD Cytofix/Cytoperm™ Plus Permeabilization Buffer (BD Pharmingen™) and stained with Alexa Fluor^®^ 647 Mouse Anti-H2AX (pS139) antibody (BD Pharmingen™) at 5 µl/assay. After a 20-minute incubation at room temperature, staining was performed with 1 µg/mL DAPI (BD Pharmingen™) diluted in 1% BSA/PBS Sigma-Aldrich). An assay was performed using the BD™ LSR II flow cytometer system and FACSdiva software. Each experiment was performed on different cell batches.

### RNA isolation and cDNA synthesis

According to the manufacturer’s instructions, total RNA was isolated from Caco-2 cells grown on T-75 flasks, using the RNeasy Mini Kit and protocol provided by the manufacturer (Qiagen, TX, USA) with DNaseI treatment. The RNA’s concentration and purity were assessed using µDrop™ Duo Plates in a Multiskan SkyHigh microplate reader (Thermo Scientific, DE, USA). RNA samples from three biological replicates were prepared from control Caco-2 cells and cells treated with 100 µg/mL PNPs. Each sample (1 µg RNA) was subjected to reverse transcription using the iScript cDNA Synthesis Kit from Bio-Rad, employing RNase H + MMLV reverse transcriptase in a final reaction volume of 20 µl, as specified in the kit’s instructions.

### qPCR assay

The expression of genes was assessed in two Caco-2 cell lines (untreated vs. nanoparticle PNPs-100 µg/mL treated) using a PrimePCR assay with a custom-designed SYBR plate (Bio-Rad), following the manufacturer’s protocol. This custom plate targeted 14 genes involved in the DNA damage response (DDR): *PARP3*,* OGG1*,* LIG3*,* LIG1*,* ATM*,* ATR*,* TP53BP1*,* XRCC1*, *RAD51*,* BRCA1*,* XRCC4*,* PARP2*,* PARP1*, and *H2AFX*, with *ACTB* serving as the reference gene. The qPCR reaction utilized 1 µl of cDNA, iTaq™ Universal SYBR^®^ Green Supermix (Bio-Rad), and primers preloaded in a 96-well plate, following the protocol for custom SYBR Green^®^ reaction setups. The qPCR experiments were performed using a CFX Opus 96 Real-Time PCR System (Bio-Rad), and gene expression data were analyzed with CFX Manager™ Software (Bio-Rad). Controls included a PCR performance test and a reverse transcription control assay (RT) to verify the corresponding stages of the experiment. The average ΔCt for the control group (derived from untreated Caco-2 cells) was used as a calibrator, with ΔΔCt calculated by subtracting this value from each ΔCt value. Fold change was then determined using the standard Eq. 2^-(ΔΔCt) as described previously. Gene expression for each gene was assessed based on three biological replicates.

### Statistical analysis

All experiments were performed in at least three independent biological replicates to confirm reproducibility. Results were displayed as mean ± SEM by Prism 4 (GraphPad Software Inc). One-way ANOVA was used to analyze experimental data. Dunnett’s post-hoc test was used for multiple comparisons of trypan blue exclusion assay, clonogenic assay, and ROS measurement, where multiple concentrations were compared against the control. P-values were considered significant: *p ≤ 0.05, **p ≤ 0.01, ***p ≤ 0.001 and ****p ≤ 0.0001.

## Results

### The effect of PNPs exposure on Caco-2 cells viability

**Fig. 1:**
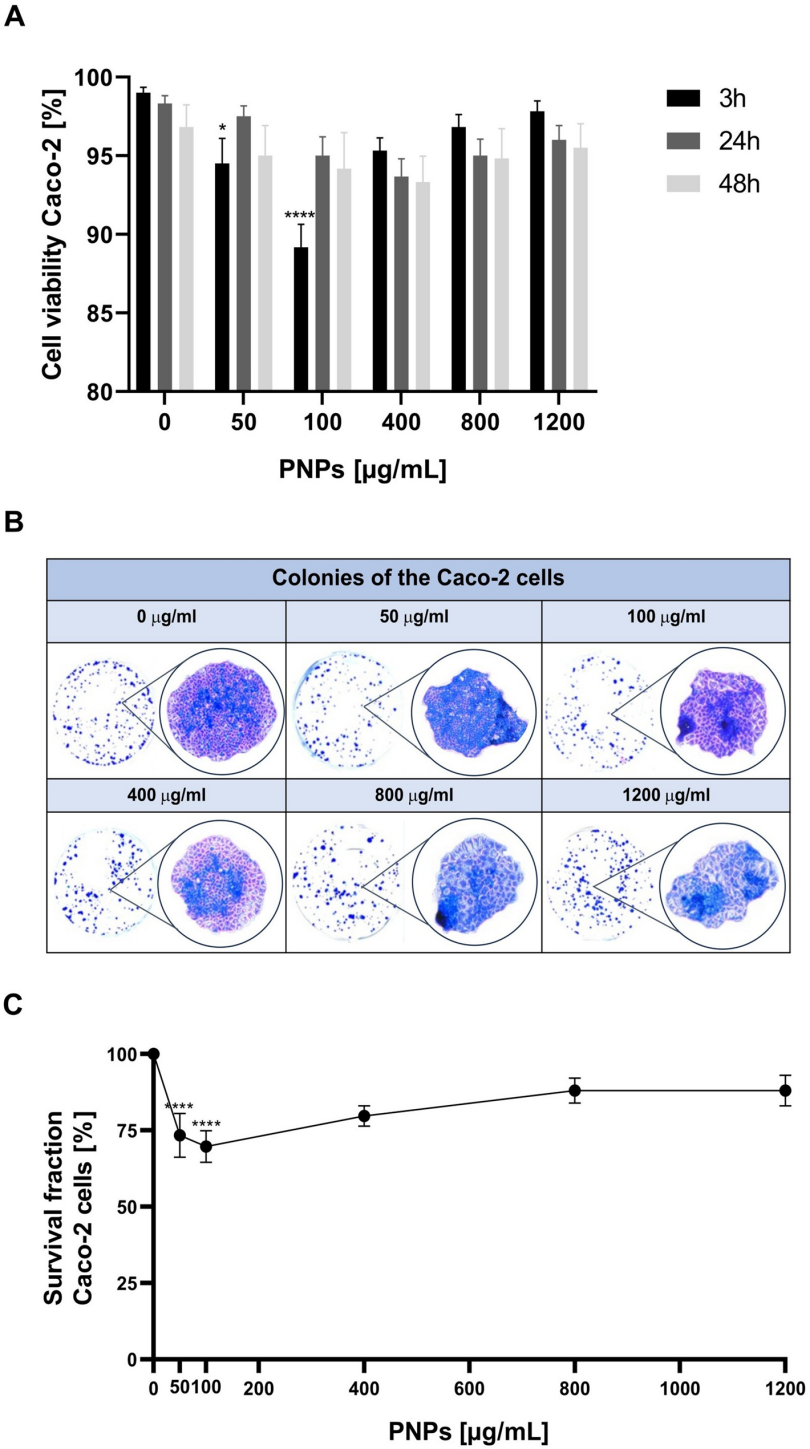
Cell viability and survival fraction after exposure of Caco-2 cells to PNPs (0, 50, 100, 400, 800, and 1200 µg/mL). **A**: Trypan blue exclusion test after 3 h, 24 h, and 48 h incubation with PNPs. Data were expressed as a percentage; the bars correspond to the mean ± SEM (*n* = 6). **B**: The colony formation assay: representative images of colonies stained with Coomassie blue for nontreated and after 24-hour treatment of cells with PNPs. **C**: The panel represents decreased colony survival fractions of Caco-2 cells exposed to PNPs compared to the unexposed cells. The colonies were analyzed after 9 days of incubation in 5% CO_2_ and 37 °C and counted using countPHICS software. Survival Fraction (SF) was considered using the formula SF = (plating efficiency of tested cells/plating efficiency of control cells x 100%). Data were expressed as a percentage; the bars correspond to the mean ± SEM (*n* = 3). P-values were considered significant: *p ≤ 0.05, **p ≤ 0.01, ***p ≤ 0.001 and ****p ≤ 0.0001

To explore the in vitro impact of PNPs exposure, we assessed the viability in Caco-2 cells at PNPs concentrations of 0 (control), 50, 100, 400, 800, and 1200 µg/mL using the trypan blue exclusion assay. The use of PNP concentrations at 50, 100, 400, 800, and 1200 µg/mL is justified based on their relevance in studying biological interactions, environmental impacts, toxicological assessments, and material property optimizations. These concentrations cover a broad range, allowing for a comprehensive evaluation of the effects and applications of PNPs [8, 17–19]. Following initial dose-setting trials, our observations revealed that cells exposed to 100 µg/mL demonstrated the most significant decrease in viability, dropping to 89% after a 3-hour incubation. This was the earliest response, with viability slightly recovering at the 24-hour and 48-hour intervals (Fig. 1A). Higher concentrations up to 1200 µg/mL did not show any effect, suggesting that the lack of a further increase may be due to nanoparticle agglomeration, which could reduce their bioavailability and interaction with cells.

We further employed the clonogenic assay to observe the long-term effects of acute exposure of PNPs (Fig. 1B and C). While the clonogenic assay primarily evaluates cell survival and proliferative capacity after exposure to a given treatment, it is indeed one of the most widely used methods for assessing long-term cytotoxic effects in toxicological studies. The ability of a single cell to form a colony reflects its capacity to undergo multiple divisions, making this assay particularly relevant for detecting delayed or long-term consequences of toxic exposure, including DNA damage or metabolic stress that may not be immediately evident in short-term viability assays. Exposure to 50 µg/mL of PNPs resulted in a survival fraction (SF) of 73% ± 9%, indicating a substantial decrease in the number and health of colonies compared to the untreated control (****p ≤ 0.0001). At a 100 µg/mL concentration, the SF was further significantly reduced to 69% ± 7%, underscoring a pronounced cytotoxic effect at this dose (****p ≤ 0.0001). This concentration of PNPs with 24-hour incubation time was taken for further studies. At 400 µg/ml, the SF was measured at 79% ± 4%, reflecting a moderate cytotoxic impact compared to lower doses. Higher concentrations of 800 and 1200 µg/mL yielded survival fractions of 87% ± 5% and 87% ± 7% respectively.

### The quantification of apoptosis dependent on PARP-1 cleavage has shown no effect on the level of apoptotic cells

**Fig. 2:**
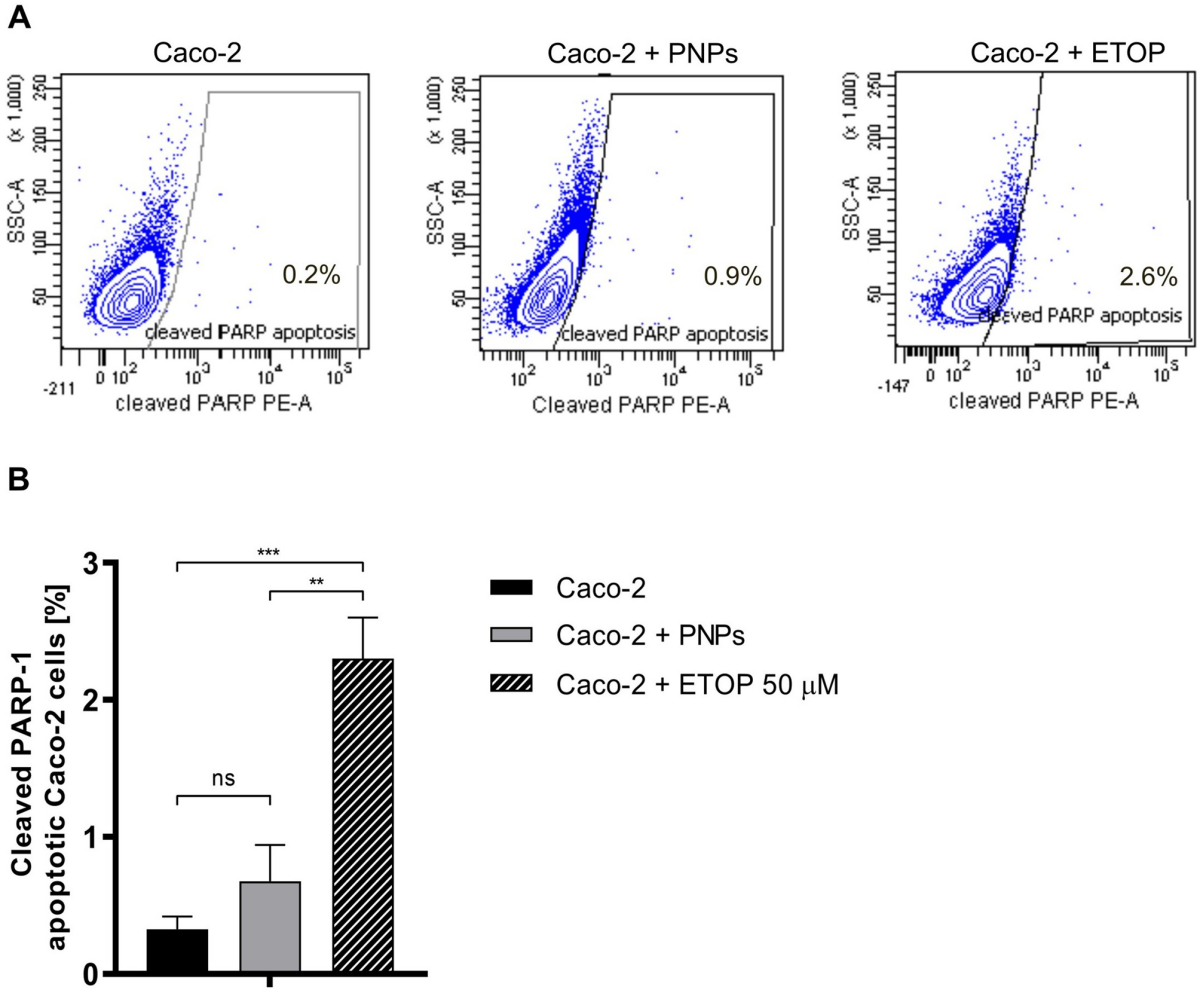
Caco-2 PNPs exposure (100 µg/mL, 24 h) on cleaved PARP-1 apoptosis. **A**: Multicolor flow cytometric representative images of Cleaved PARP (Asp214) PE versus BrdU PerCP-Cy™5.5 profile. **B**: Data were expressed as a percentage; the point bars represent the mean ± SEM (*n* = 4) for apoptosis. 2-hour incubation with etoposide (ETOP), was used as a positive control with a final concentration of 50 µM. The fragment of cleaved-PARP1 (89 kDa) was used as a marker of apoptosis (PE mouse anti-cleaved PARP (Asp214) antibody). P-values were considered significant: *p ≤ 0.05, **p ≤ 0.01, ***p ≤ 0.001 and ****p ≤ 0.0001

We assessed apoptosis assay by flow cytometry using an Anti-PARP-1 antibody. During apoptosis, caspase-3 cleavage of poly(ADP-ribose) polymerase 1 (PARP-1) results in PARP-1 inactivation and the consequent inability of cells to repair DNA damage. Consequently, the appearance of the 89 kDa cleaved fragment of PARP is a dependable indicator of cellular apoptosis, providing a measurable link between DDR processes and apoptosis through biochemical changes [20]. A concentration of 100 µg/mL of PNPs was selected after a 24-hour incubation based on the results from the clonogenic assay. Figure 2A and B show that after 24-hour incubation of the Caco-2 cell line with PNPs at this concentration, the percentage of apoptotic cells did not change significantly (0.68% ± 0.4% compared to control cells 0.33% ± 0.14%) In contrast, treatment with the positive control 50 µM etoposide (ETOP) led to a significant increase in apoptosis, reaching 2.3% ± 0.15%, confirming the sensitivity of the assay.

### PNPs exposure does not affect cell cycle phases

**Fig. 3:**
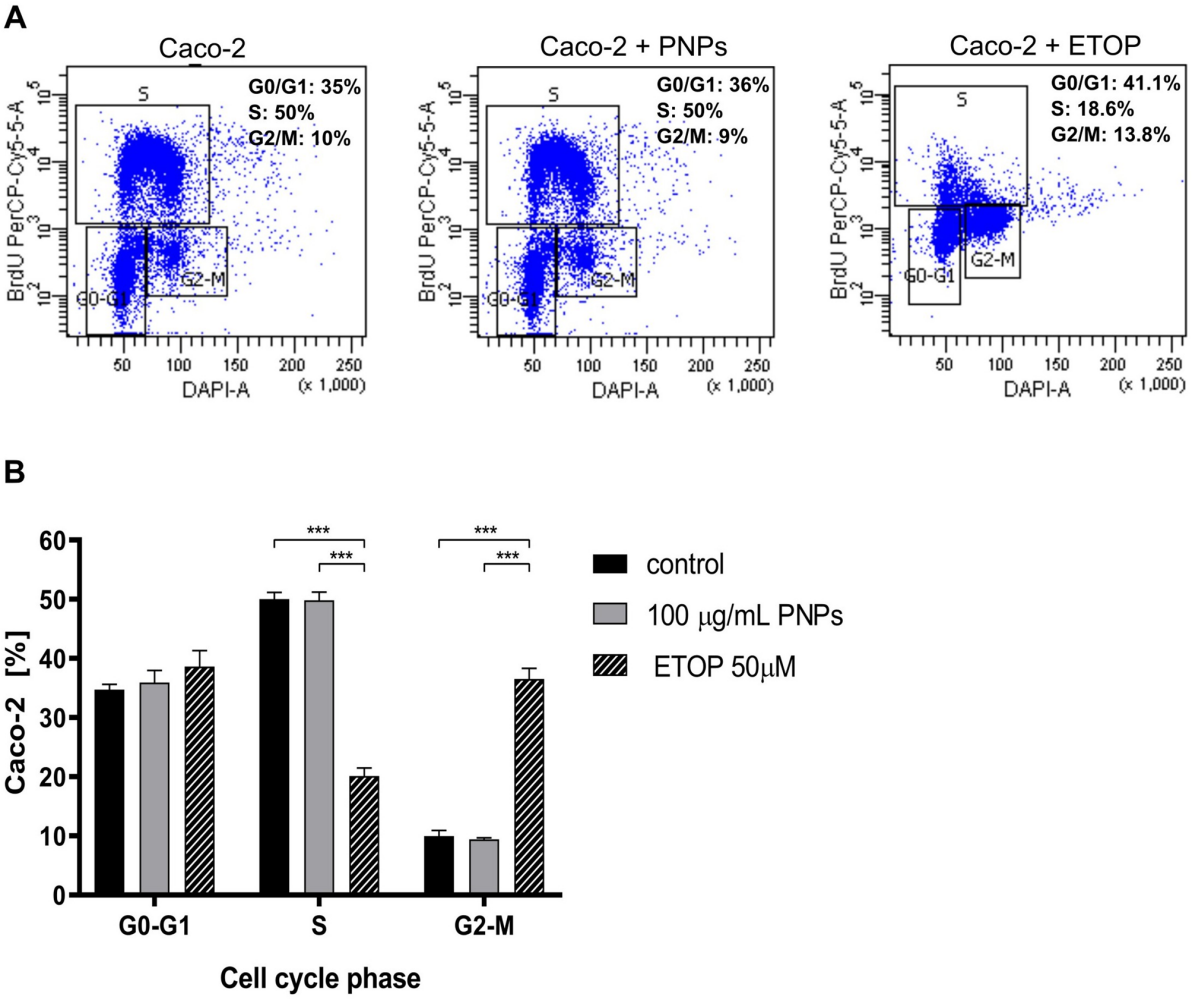
Flow cytometry identification of cell cycle changes with anti-BrdU antibody in Caco-2 cells after PNPs exposure (100 µg/mL, 24 h). 2-hour incubation with etoposide (ETOP), was used as a positive control with a final concentration of 50 µM. The cells were labeled with 10 µM BrdU for 1 h and stained with BrdU PerCP-Cy™5.5 antibody. Flow cytometry representative images (**A** panel) of DAPI versus BrdU PerCP-Cy™5.5 staining profile (% of Caco-2 cells in each phase) and relative quantification (**B** panel). Cells were gated correctly: G0/G1 phase, S phase, and G2/M phase. Data were demonstrated as percentages of cells and the bars correspond to the mean ± SEM (*n* = 4). P-values were considered significant: *p ≤ 0.05, **p ≤ 0.01, ***p ≤ 0.001 and ****p ≤ 0.0001

To assess if alterations in the cell cycle correlate with the observed reduction in cell survival following treatment with 100 µg/mL of PNPs, we carried out a cell cycle analysis after 24 h of incubation. Exposing cells to BrdU enables the incorporation of BrdU by actively dividing cell populations. Based on Fig. 3A and B, analysis of the cell cycle distribution of Caco-2 induced by PNPs showed no significant changes in cell cycle phases. In the G0/G1 phase, 34.73% ± 1.33 cells untreated were recorded, while by 24-hour incubation with the PNPs, the number of cells in this phase did not significantly change (35.93% ± 3.38) (Caco-2 + PNPs). The average proportions of cells in the S phase were 50% ± 1.7% and 49.8% ± 2.2% for Caco-2 and Caco-2 + PNPs cells, respectively. No significant differences were shown (Fig. 3B). No significant changes were also observed in the G2/M phase, where the average cell proportions were 9.97% ± 1.3% and 9.45% ± 0.3% for Caco-2 and Caco-2 + PNPs cells, respectively. In contrast, treatment with the positive control 50 µM etoposide (ETOP) resulted in a clear accumulation of cells in the G2/M phase, with an average proportion of 36.53% ± 1.03%, while the G0/G1 and S phases were reduced to 38.6% ± 1.45% and 20.13% ± 0.77%, respectively. This confirms that the assay is capable of detecting cell cycle perturbations when present, further supporting the conclusion that PNP exposure does not lead to significant cell cycle arrest.

### PNPs induce oxidative stress in Caco-2 cells

**Fig. 4:**
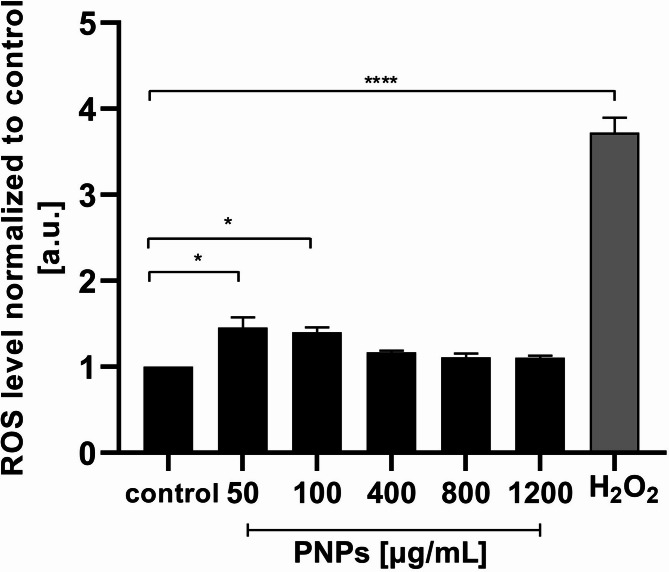
Caco-2 PNPs exposure (0, 50, 100, 400, 800, and 1200 µg/mL) on intracellular ROS levels. 1.5 mM H₂O₂ was used as a positive control, [a.u.] = arbitrary units. The data was normalized to the control and presented as mean ± SEM (*n* = 3) for ROS level analysis (H2DCFDA probe). P-values were considered significant: *p ≤ 0.05, **p ≤ 0.01, ***p ≤ 0.001 and ****p ≤ 0.0001

To check the probable production of reactive oxygen species by Caco-2 cells in the presence of PNPs at different concentrations (0, 50, 100, 400, 800, 1200 µg/mL, after 24 h of incubation), DCFDA fluorescence probe staining was performed, allowing the determination of ROS levels. Figure 4 shows the results normalized to controls and expressed as arbitrary units. After applying 50 and 100 µg/mL PNPs, statistically significant results were obtained (*p ≤ 0.05). An increase in reactive oxygen species levels was observed in these samples, respectively: 1.45 ± 0.14 and 1.4 ± 0.08. At higher concentrations of PNPs (400, 800, and 1200 µg/mL), there is no significant increase in the level of ROS. To validate the sensitivity of the ROS assay, we included 1.5 mM H₂O₂ as a positive control, which resulted in a significant increase in ROS levels (normalized mean: 3.73 ± 0.21 a.u., ****p ≤ 0.0001), confirming the reliability of detection method.

### PNPs exposure does not induce DNA damage– both SSBs and DSBs

**Fig. 5:**
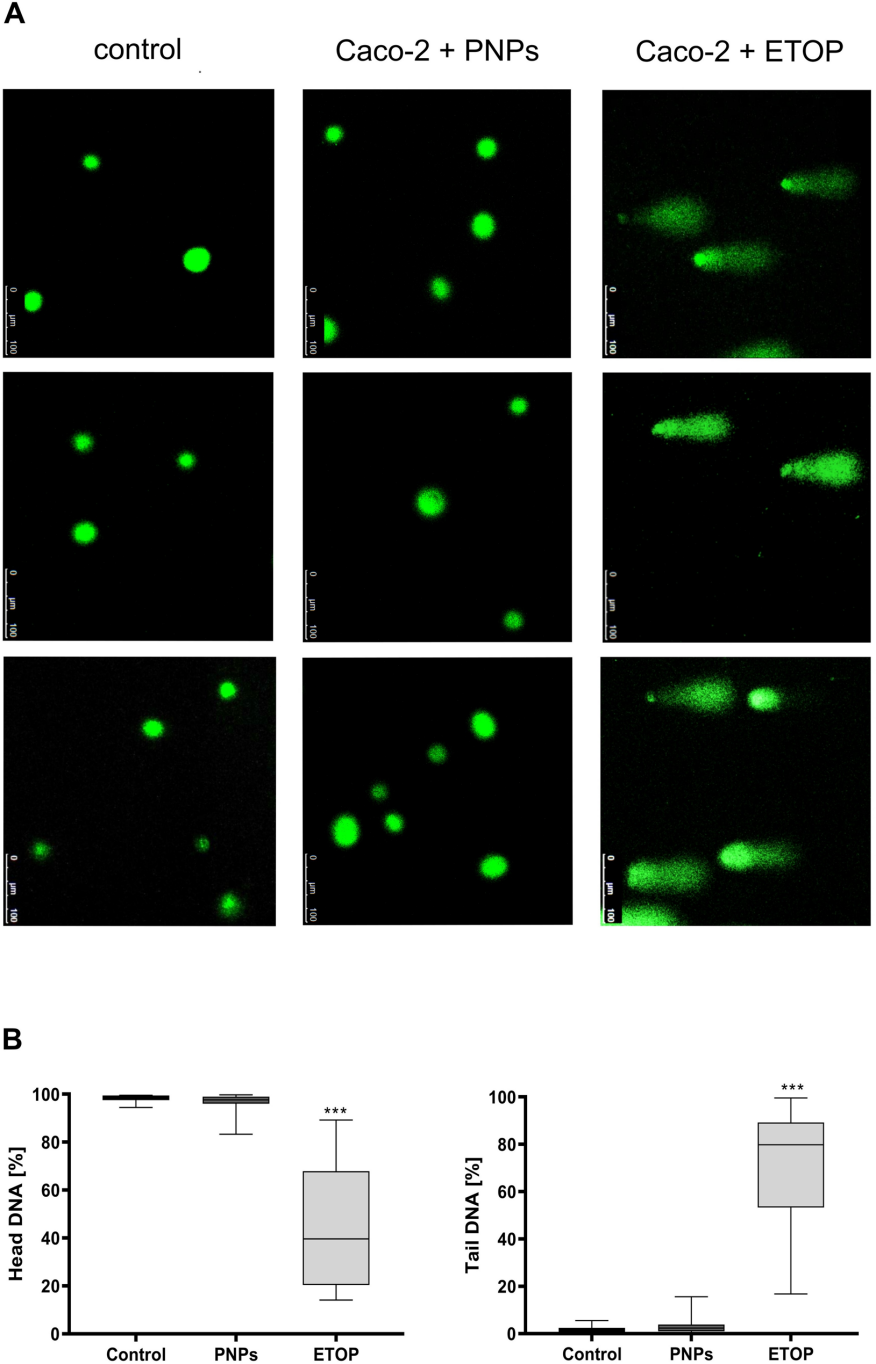
Identification of SSBs and DSBs in Caco-2 cells after PNPs exposure (100 µg/mL, 24 h) with alkaline comet assay. 50 µM etoposide (2 h incubation) was used as a positive control. **A**: Representative comets with a comet tail (fragmented DNA) and a head (intact DNA), visualized under 40x immersion oil, confocal microscope Leica SP5 with SYBR SAFE™ staining using fluorescein filter with maximum excitation/emission 496 nm/522 nm). **B**: Quantification of comet assay images was performed using OpenComet v1.3.1 software for % of DNA in the head and % of DNA in the tail (box plots). Results are based on the analysis of at least 50 comets per replicate (*n* = 3). P-values were considered significant: *p ≤ 0.05, **p ≤ 0.01, ***p ≤ 0.001 and ****p ≤ 0.0001

The finding that PNPs induce oxidative stress in cells of the Caco-2 line indicates an essential molecular mechanism involved in these nanoparticles’ toxicity. Therefore, the next step in the study was to assess the potential DNA damage induced by these nanoparticles. An alkaline comet assay was used to examine the level of DNA damage, single- (SSBs), and double-strand DNA breaks (DSBs) in cells exposed to the PNPs. The alkaline comet assay is a “gold standard” method widely used in genotoxicity and biomonitoring studies, including base changes, DNA cross-links, drug development, and alkali-sensitive sites [21]. Analyzing the shape and migration pattern of the DNA “comet tail” enables the detection and evaluation of DNA damage. Caco-2 cell line cells cultured under standard conditions constituted the negative control sample. Cells after 24-hour exposure to PNPs at a concentration of 100 µg/mL comprised the test sample. A positive control, 50 µM etoposide (ETOP), was also used to test the method’s efficacy. Positive control cells showed significant DNA damage, observed in numerous replicates (Fig. 5A, representative images). Clear comet “heads” with long “tails” were observed, indicating significant DNA fragmentation (Fig. 5B, ***p ≤ 0.001). This result confirms the efficiency and sensitivity of the alkaline comet assay. No comet “tails” were observed in untreated and PNPs-treated cells (Fig. 5A and B), indicating the absence of DNA damage in the control and experimental samples.

**Fig. 6:**
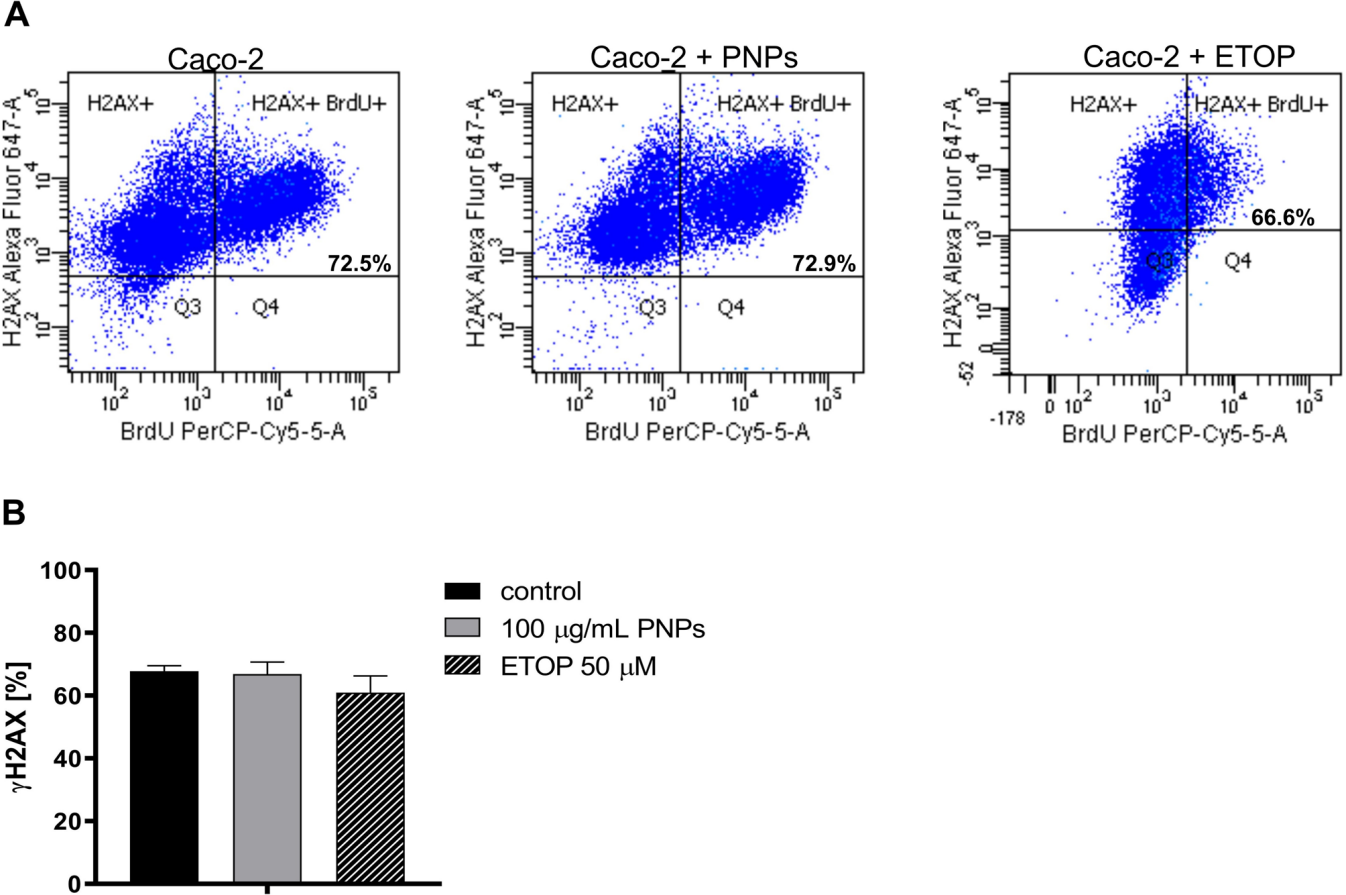
Flow cytometry identification of DNA double-strand breaks in Caco-2 cells after PNPs exposure (100 µg/mL, 24 h). 2-hour incubation with etoposide (ETOP), was used as a positive control with a final concentration of 50 µM. **A**: Flow cytometry representative images of BrdU PerCP-Cy™5.5 and H2AX (pS139) Alexa Fluor^®^ 647 (% indicating the proportion of DSBs in the presented experiment). **B**: The distribution of γH2AX in Caco-2 cells (in control and PNPs-treated Caco-2 cells); the bars correspond to the mean ± SEM (*n* = 4). P-values were considered significant: *p ≤ 0.05, **p ≤ 0.01, ***p ≤ 0.001 and ****p ≤ 0.0001

As there was no evidence of DNA damage, the next step was to quantify the data obtained on the population of cells stained with the marker for γH2AX, which allows the assessment of DNA double-stranded break lesions [22, 23]. Exposure of the Caco-2 cell line to PNPs at a concentration of 100 µg/mL for 24 h showed no significant changes in the level of γH2AX phosphorylation on serine 139. Staining with Alexa Fluor™ 647 Mouse Anti-H2AX antibody (pS139) showed that the average number of γH2AX foci was 67.77% ± 2.4% and 66.9% ± 6.4% for Caco-2 and Caco-2 + PNPs cells, respectively (Fig. 6A and B). Statistical significance was not demonstrated. In contrast, treatment with the positive control etoposide resulted in γH2AX foci levels, reaching 60.97% ± 3.76%. However, the proportion of γH2AX-positive cells following etoposide treatment appears lower than expected compared to the untreated Caco-2 control. This could be attributed to the high basal γH2AX levels observed in Caco-2 cells, which may result from endogenous replication stress, chromatin remodeling, or a high rate of background signaling in this cell line. Such intrinsic factors may mask the relative increase induced by etoposide, leading to a seemingly moderate fold change despite its well-established role as a potent inducer of DNA double-strand breaks. The obtained results suggest that the 100 µg/mL PNPs do not induce the formation of the DNA double-strand breaks.

### PNPs exposure induced Inhibition of DNA damage response

**Fig. 7:**
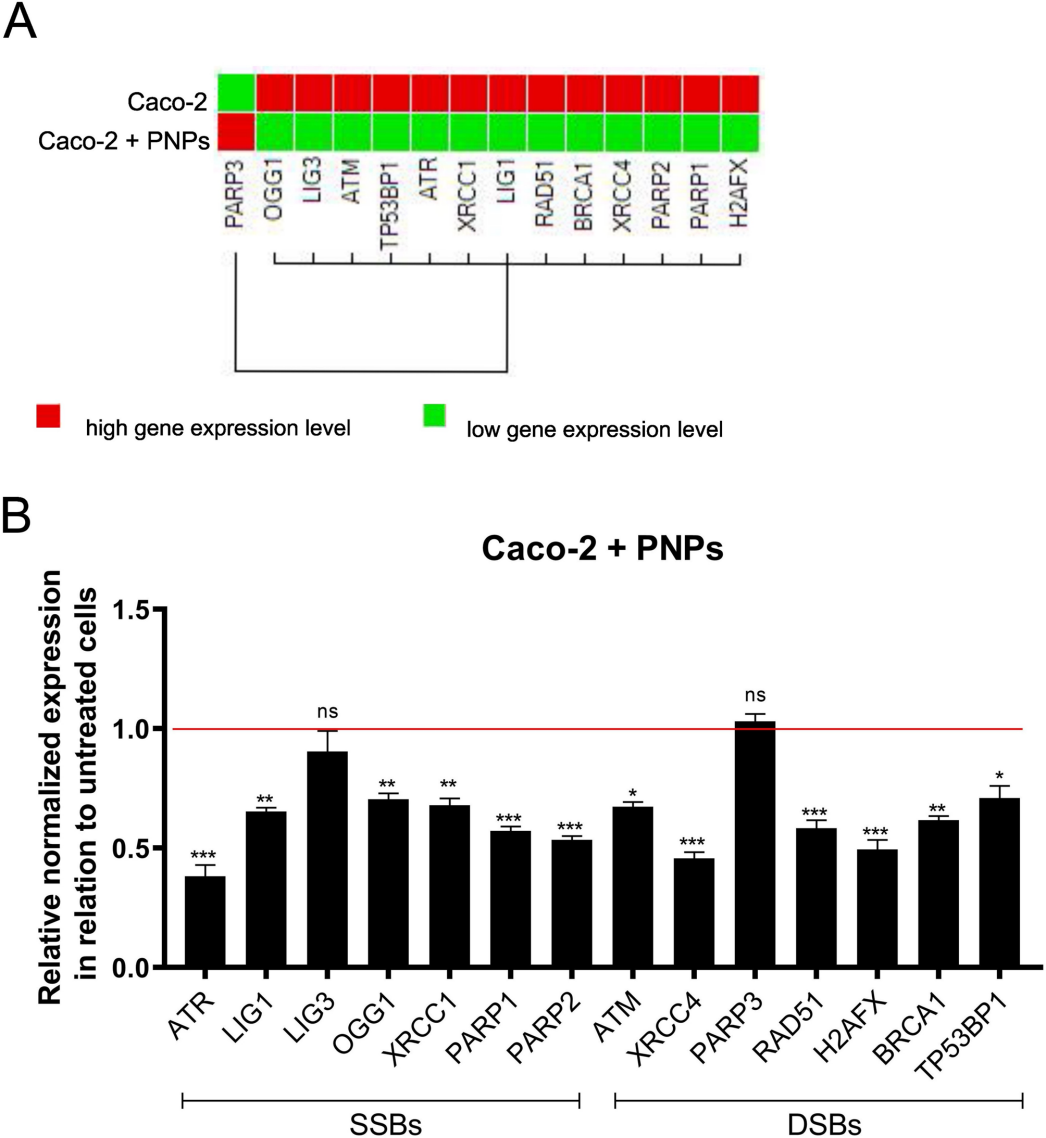
Real-time PCR analysis of the normalized expression of DDR signaling genes after PNPs exposure (100 µg/mL, 24 h). **A**: Cluster heatmap, red signifies a relatively high gene expression level, while green signifies a low level. The data were analyzed (*n* = 3) and clustered by targets using the Reference Gene Selection Tool from Bio-Rad CFX Maestro software. **B**: Bar plot of the relative expression of all genes (belonging to SSBR and DSBR) calculated *via* the ΔΔCt method. The bars correspond to the mean ± SEM (*n* = 3). P-values were considered significant: *p ≤ 0.05, **p ≤ 0.01, ***p ≤ 0.001 and ****p ≤ 0.0001

In this study, Caco2 cells were exposed to 100 µg/mL of PNPs to assess the impact on DNA damage response (DDR) pathways. We utilized qPCR arrays to examine the expression levels of 14 essential DDR genes, with the actin beta (*ACTB*) housekeeping gene as the reference gene. After 24 h of exposure to PNPs, there was a significant decrease in the expression of almost all studied DDR genes compared to the unexposed control cells (Fig. 7A– heatmap and 7B relative normalized expression), except for *PARP3* (poly (ADP-ribose) polymerase 3), observed a slight upregulation. *OGG1* (8-oxoguanine DNA glycosylase), *LIG1* (DNA ligase 1), *LIG3* (DNA ligase 3), *PARP1* (poly (ADP-ribose) polymerase 1), *PARP2* (poly (ADP-ribose) polymerase 2), and *XRCC1* (X-ray repair cross-complementing protein 1) are all key in DNA single-strand breaks repair (SSBR/BER). Their reduced expression could impair these repair processes. *ATM* (ataxia telangiectasia mutated), *ATR* (ATM and Rad3 related), *BRCA1* (breast cancer 1), *RAD51* (RAD51 recombinase), *TP53BP1* (tumor protein p53 binding protein 1), *XRCC4* (X-ray repair cross-complementing protein 4), and *H2AFX* (H2A histone family member X) are crucial for DNA double-strand breaks repair (DSBR). Their decreased expression suggests potential vulnerabilities in the cell’s repair of double-strand breaks.

Obtained results suggest that exposure to polystyrene nanoparticles significantly suppresses critical pathways involved in single-strand and double-strand DNA break repair, underscores potential threats to genomic stability, and highlights potential risks associated with nanoplastics exposure in intestinal cells.

## Discussion

Plastics, particularly nanoplastics, pose significant risks to living organisms, including humans [1, 8, 24–28]. Numerous studies focus on the adverse effects of nanoplastics, especially polystyrene nanoparticles, on various organisms, including aquatic species, nematodes, and neuronal and gastrointestinal cells. These nanoparticles have been shown to accumulate in marine organisms, such as mussels and fish, causing oxidative stress and neurotoxicity [29, 30]. In zebrafish (*Danio rerio*), exposure to polystyrene nanoplastics triggers developmental toxicity by activating the BER and oxidative stress response pathways [6]. In nematodes like *Caenorhabditis elegans*, nanoplastics have been observed to induce Parkinson’s disease, circadian rhythm, and transgenerational toxicity [31–33]. Additionally, in human neural stem cells, polystyrene nanoplastics have been shown to impact molecular pathways, potentially disrupting neurogenesis and neuronal function [34]. The gastrointestinal tract, particularly human intestinal Caco-2 cells, is often used as a model to study nanoparticle translocation and toxicity [35, 36]. Caco-2 cells are widely recognized and utilized as an in vitro model for studying the intestinal barrier due to their ability to mimic the characteristics of human intestinal epithelial cells [37, 38]. Caco-2 cells, derived from human colon carcinoma, spontaneously differentiate into polarized cells that exhibit morphological and biochemical features of small intestinal enterocytes, including the formation of brush borders and tight junctions [39]. This differentiation is crucial for studying the intestinal barrier’s function and integrity. The Caco-2 cell model is extensively used to evaluate the permeability and transport mechanisms of drugs and chemicals across the intestinal barrier. The permeability values obtained from Caco-2 cells correlate well with human in vivo absorption data, making them a reliable tool for predicting oral drug absorption [40].

Several studies have reported varying systemic levels of nanoplastics depending on exposure routes, particle characteristics, and detection methods. While the exact physiological concentrations remain difficult to determine due to methodological limitations in quantifying nanoplastics in biological systems, previous reports indicate that nanoplastic concentrations in human tissues and fluids may range from ng/mL to µg/mL, particularly in individuals with chronic environmental exposure [1, 41, 42]. Our selected concentration range (50–1200 µg/mL) and 24-hour incubation time were chosen to encompass both physiological and supra-physiological exposure scenarios as previously reviewed in detail [1, 8]. The lowest concentration (50 µg/mL) represents a low exposure level to assess minimal biological and environmental impacts [1, 8, 43]. 100 µg/mL is commonly used in environmental and toxicological studies, making it a relevant dose for comparison with existing literature [1, 24, 25, 43–45]. 400 µg/mL was selected as an intermediate concentration to observe potential dose-dependent effects. 800 µg/mL represents a higher concentration at which significant biological and material property changes may become apparent [8]. Finally, 1200 µg/mL serves as the upper limit to assess the maximum impact and potential risks of nanoplastic exposure [8]. This concentration range ensures both biological relevance and a comprehensive assessment of dose-dependent effects, aligning with previously published in vitro studies on nanoplastic toxicity.

Our research indicates that nanoplastics have minimal toxic effects on the Caco-2 cell line under experimental conditions. For instance, cytotoxicity was relatively low, with a 100 µg/mL concentration reducing cell viability by only 11%. This result contrasts with macrophages, where the same concentration caused a 30% reduction in viability, and in A549 cells, where 400 µg/mL reduced viability to 55% [46]. This suggests that Caco-2 cells, as part of the primary barrier in the human body, may exhibit resistance to nanoplastics-induced cytotoxicity, consistent with the studies demonstrated before [24, 25]. Furthermore, the size and surface charge of the PNPs are critical factors in toxicity. Modified PNPs with diameters of 20 and 40 nm and surface charges (-COOH and -NH_2_) significantly reduced cell viability after just four hours of incubation [47]. Clonogenic assays showed a reduction in Caco-2 cell survival, starting at 50 µg/mL, with survival dropping to 69% ± 7% at a 100 µg/mL concentration. Higher concentrations led to a smaller decrease in survival, potentially due to particle agglomeration and decreased cellular uptake [4].

Oxidative stress, another concern associated with nanoplastics, is linked to generating reactive oxygen species. Our study demonstrated a statistically significant rise in ROS levels after 24-hour incubation with PNPs at 50 and 100 µg/mL concentrations. However, ROS levels did not increase significantly at higher concentrations (400, 800, 1200 µg/mL), likely due to nanoparticle agglomeration [4]. Other studies using the DCFDA probe for measuring ROS in Caco-2 cells found inconsistent results, with some failing to observe oxidative stress induction [48]. The genotoxicity of nanoplastics was evaluated using the comet assay and γH2AX marker to detect DNA strand breaks. In this study, the comet assay produced negative results for Caco-2 cells exposed to 100 µg/mL of PNPs, aligning with previous studies’ findings [24]. In contrast, some studies have reported positive comet assays with other cell lines, such as HepG2 and THP-1, when exposed to modified PNPs with different surface chemistries [49, 50]. These findings suggest that surface charge and nanoparticle chemistry are pivotal in genotoxic potential. The γH2AX assay also showed no significant DNA damage in Caco-2 cells, but further research is needed to confirm the broader implications of nanoplastic exposure on DNA integrity [51]. The high levels of H2AX phosphorylation can be explained as follows. Caco-2, exhibit a high baseline level of γH2AX due to normal cellular processes, such as replication stress, transcriptional activity, and endogenous oxidative stress [52]. γH2AX is a marker for DNA double-strand breaks, which can occur during DNA replication [22]. The phosphorylation of H2AX is particularly noted in the S-phase of the cell cycle, indicating its association with replication stress. High transcriptional activity can lead to DNA damage and subsequent γH2AX formation. Moreover, endogenous oxidative stress contributes to the baseline levels of γH2AX in Caco-2 cells [53]. Therefore, detecting treatment-induced increases in γ-H2AX in Caco-2 cells can be challenging, as the high background signal may obscure subtle effects. To address this, we complemented γ-H2AX analysis with functional cell cycle data. Our cell cycle analysis demonstrated that etoposide clearly induced DNA damage and checkpoint activation in Caco-2 cells, as evidenced by pronounced G2/M arrest and reduction of the S-phase population. Moreover, in our parallel studies using HBE wt and HBE ΔαBK_Ca_ cells [54], we confirmed a robust γ-H2AX response to etoposide, validating the sensitivity and performance of our assay. Finally, the absence of G2/M arrest following PNP treatment further supports the conclusion that PNPs do not induce significant DNA double-strand breaks under the tested conditions. Therefore, we believe that combining the alkaline comet assay with γ-H2AX detection strengthens the validity of our findings, especially in a model such as Caco-2 where reliance on γ-H2AX alone may lead to inconsistent results.

While short-term studies indicate minimal cytotoxicity and genotoxicity in Caco-2 cells, long-term exposure to nanoplastics remains a concern. However, it was also shown that long-term exposure to PNPs does not result in DNA damage/oxidative stress using the Caco-2 cell line model [24]. Accumulation in tissues and chronic exposure could lead to more severe health effects, potentially contributing to carcinogenesis through mechanisms like oxidative stress and DNA damage [55].

Our study demonstrated that exposure to PNPs in Caco-2 cells led to the downregulation of several critical DDR genes, particularly those involved in SSBR and DSBR. Genes such as *OGG1*,* LIG1*,* LIG3*,* PARP1*, and *XRCC1*, all vital for the base excision repair (BER) pathway, were downregulated, suggesting a reduced capacity to repair oxidative DNA damage. This impairment could contribute to genomic instability in cells exposed to PNPs over time. Inhibition of *XRCC1* was also observed after PNPs exposure on human neural stem cells. However, smaller diameters and concentrations were used (30 nm PNPs at 0.5, 2.5, and 10 µg/mL) [34]. In contrast, the study performed by Feng et al. on zebrafish embryos revealed an upregulation of critical genes associated with the oxidative stress response and DNA repair, including those involved in the BER pathway [6]. Notably, genes, such as *OGG1* and *PARP1* were found to be significantly upregulated. These genes are essential for recognizing and repairing oxidative DNA lesions. Feng et al. also observed the upregulation of genes such as *SOD1* (superoxide dismutase 1) and *CAT* (catalase), both involved in the oxidative stress response. This suggests that exposure to PNPs in zebrafish embryos triggers a protective mechanism to counteract oxidative damage through ROS detoxification and DNA repair activation [6]. Differences in organismal context and cellular types may explain the contrasting results between our study and Feng et al. Zebrafish embryos, as rapidly developing organisms, may activate more robust oxidative stress responses and repair mechanisms, possibly to protect against developmental abnormalities. On the other hand, Caco-2 cells, which are differentiated intestinal cells, might have a limited capacity to mount a similar protective response when exposed to nanoplastics. Our study’s downregulation of *OGG1*,* PARP1*, and *XRCC1* suggests that PNPs exposure may impair essential DNA repair pathways in adult human intestinal cells, potentially leading to long-term genomic instability if exposure persists. Additionally, Feng et al. reported that the activation of these pathways in zebrafish was associated with developmental toxicity, implying that the upregulation of DNA repair genes is a response to counteract potential damage during critical developmental stages [6]. In contrast, the diminished response in Caco-2 cells may indicate that differentiated cells, particularly those with barrier functions, have a reduced ability to manage nanoplastics-induced stress, significantly over more extended exposure periods. Additional molecular studies are necessary to fully understand the impact of nanoplastics exposure, particularly under long-term or chronic conditions.

## Conclusions

This study examines the sublethal effects of polystyrene nanoparticles on intestinal barrier cells, emphasizing the risks of chronic exposure to these contaminants. It suggests that such exposure can lead to genome instability and impair the cells’ ability to repair oxidative DNA damage, posing potential long-term health risks.

## Data Availability

All data generated or analyzed during the current study are included.

## Abbreviations

ATM: Ataxia telangiectasia mutated kinase
ATR: ATM and Rad3 related kinase
BER: Base excision repair
BRCA1: Breast cancer 1
DDR: DNA damage response
DSB_S_: Double-strand breaks
H2AFX: H2A histone family member X
LIG1: DNA ligase 1
LIG3: DNA ligase 3
OGG1: 8-oxoguanine DNA glycosylase
PARP: Poly(ADP-ribose) polymerase
PARP1: Poly (ADP-ribose) polymerase 1
PARP2: Poly (ADP-ribose) polymerase 2
PARP3: Poly (ADP-ribose) polymerase 3
PNP_S_: Polystyrene nanoparticles
RAD51: RAD51 recombinase
SSBs: Single-strand breaks
TP53BP1: Tumor protein p53 binding protein 1
XRCC1: X-ray repair cross-complementing protein 1
XRCC4: X-ray repair cross-complementing protein 4
